# Prof. Eve’s Card

**Published:** 1851-01

**Authors:** 


ARTICLE VII.

*Surgical Report for the American Medical Association.*

The committee is invited to meet in the Charleston Hotel, South Carolina, the evening of the first Tuesday in May next. All professional brethren, who have *surgical* facts connected with the improvement of this branch of the profession during the year, will please address them to the chairman of the committee by the first of April, at Augusta, Georgia. As all cannot be reached by a circular, it is hoped no one will wait for a more direct application than this general invitation.

By extending this notice, the medical periodicals of our country will advance the interest of the American Medical Association, and the editors will confer a favor upon their recent confrere, PAUL F. EVE, M. D.

Prof. of Surgery in the Louisville University, and Chairman of the Committee on Surgery, of the American Medical Association.

Louisville, Ky., Dec. 1850.

